# ‘The Right to Have a Family Should Not Be Determined by Your Postcode’: Rural Community Experiences With Fertility Care

**DOI:** 10.1111/ajr.70237

**Published:** 2026-07-30

**Authors:** Amanda Mackay, Selina Taylor, Emma Anderson, Beverley Glass

**Affiliations:** ^1^ Pharmacy, College of Medicine and Dentistry James Cook University Townsville Queensland Australia; ^2^ Centre for Rural and Remote Health James Cook University Mount Isa Queensland Australia; ^3^ College of Medicine and Dentistry James Cook University Townsville Queensland Australia

**Keywords:** assisted reproductive technologies, distance, infertility, IVF, remote

## Abstract

**Objective:**

Women in rural and remote Queensland face greater challenges in accessing fertility care than those residing in large regional or metropolitan centres of Australia. This study aims to explore the experiences of women accessing fertility care while living in a rural or remote area of Queensland and their perspectives on ways to improve fertility care.

**Setting:**

Interviews were conducted either in‐person (Townsville, Emerald, Cloncurry, Mount Isa), via telephone or Microsoft Teams.

**Participants:**

Persons currently or previously accessing fertility care, while living rurally in Queensland, identifying as female at birth, being fluent in the English language, and aged 18 years or older participated.

**Design:**

Qualitative semi‐structured interviews, with data analysed via reflexive thematic analysis, were followed by a descriptive content analysis of participants' ideas for improving rural fertility care.

**Results:**

Two main themes with 18 sub‐themes emerged from the data. Theme 1: experiences of participants in accessing and receiving fertility care, whilst living in a rural or remote area, and Theme 2: participants' perspectives on how to improve the fertility care experience in rural and remote areas. Residence in a rural or remote location added to the complexity of fertility‐related experiences. Rural living influenced fertility knowledge and information access, the accessibility of services, fertility care choice, privacy and social support. Participants articulated strategies from the availability of local information hubs to policy changes aimed at mitigating fertility care postcode‐related disadvantage.

**Conclusion:**

Rural and remote persons accessing fertility care have highlighted not only the challenges experienced but have proposed strategies to improve rural fertility care.

## Highlights

**What is already known on this subject?:**

- Access to fertility clinics to receive ART is difficult for those in rural and remote areas due to most being in large regional or metropolitan areas.
- Services at large regional fertility clinics may be less than what is available in metropolitan areas.

**What this paper adds?:**

- Providing access to fertility care in the broader sense for people in rural and remote areas, not just ART.
- Focusing on rural and remote, and not large regional populations.
- Examining fertility care information currently available to rural and remote individuals and identifying gaps to support improvements in service delivery.
- Patient perspectives on strategies to improve fertility care in rural and remote areas.

## 1 Introduction

Existing disparities in access to medical care between rural and remote communities and metropolitan Australia are also evident among individuals experiencing fertility challenges [1, 2]. Rural health service challenges such as limited resources, gender‐insensitive or judgmental care, time‐pressured services and concerns regarding privacy further restrict access [3]. These are compounded by broader issues, including socio‐cultural norms, insufficient funding for rural health services and long distances to specialised fertility care [3, 4].

Accessing fertility care, which encompasses not only assisted reproductive technologies (ART) but also fertility awareness, support and management [5], requires persons to have knowledge of when, where, and how to seek help. A significant proportion of Australians were found to have inadequate fertility knowledge leading to decreased ability to manage fertility effectively [6]. Topics included age‐related fertility declines, negative impacts of lifestyle‐related factors such as smoking and obesity, and fertility misinformation [6, 7, 8]. Additionally contributing to limited fertility awareness is that ‘infertility’ is treated as a taboo topic and is seldom discussed [8, 9].

Although treating infertility with in vitro fertilisation (IVF) at an ART fertility clinic is relatively common, not all persons wish to undergo IVF without trying other options [10, 11]. Reasons are both complex and personal, ranging from emotional, physical and financial costs through to cultural and religious reasons [10, 11]. Dependent on the cause of infertility, other options could include lifestyle modification, fertility awareness‐based methods (FABM) or medical ovulation induction [12, 13]. However, for those who do want to access IVF or ART, the distance to ART fertility clinics often presents a challenge [4, 11]. Specialist ART fertility clinics, located primarily in metropolitan and large regional centres, often require significant travel for rural and remote residents and result in decreased utilisation rates of ART [4, 14]. Although publicly funded ART fertility clinics have increased in some states of Australia [15, 16, 17], the majority of services providing ART are private sector organisations, with commercial decisions potentially impacting the location of services and hence accessibility [1].

Recent studies documenting persons' experiences and barriers to accessing treatment when living a distance from ART fertility clinics have focussed on regional areas or access to ART fertility clinics, not fertility care [1, 18, 19]. This study aims to contribute new insight by exploring the experiences of patients who have accessed fertility care while living in a rural or remote area and their perspectives on ways to improve this care for people living in similar locations.

## 2 Methods

This study was approved by James Cook University Human Research Ethics Committee (H9580) on the 8th of October 2024.

### 2.1 Study Design

A constructivist descriptive methodology was used in this study to identify the experiences and perspectives of persons' who accessed fertility care while living in a rural or remote location of Queensland. Fertility care was considered any health service sought by a person that related to infertility prevention, diagnosis or treatment and included fertility advice, awareness, support and/or management [5, 20]. Semi‐structured interviews were conducted to capture in‐depth the experiences of participants by using open‐ended questions, allowing personal perspectives to be shared [21]. We applied reflexive thematic analysis to examine the lived experiences of fertility challenges in rural settings [22, 23]. Descriptive content analysis summarised participants' ideas for improving rural fertility [24].

### 2.2 Participants

Persons currently or previously accessing fertility care, while living in a rural or remote area of Queensland, identifying as female at birth, fluent in English language, and aged 18 years or older were eligible to participate. The study was advertised through professional networks, social media platforms (Facebook and LinkedIn) to allow for purposive with subsequent snowball sampling [25]. The recruitment flyer included a Qualtrics link to plain language information on the study's purpose, reasons for doing the research, participation requirements, an information sheet, and informed consent form. Participants could express their interest in participating by either emailing the primary investigator (AM) or completing an expression of interest form via the link. Those who expressed interest were emailed copies of the participant information sheet, informed consent form, and timeframes and locations available to schedule an interview. Interested persons were then asked for their preference of telephone, online (Microsoft Teams) or in‐person interviews.

### 2.3 Data Collection

Twenty‐four interviews were conducted by the primary investigator (AM) between May and August 2025 in‐person at four physical locations, via Microsoft Teams or by telephone using an interview guide. All participants provided written informed consent prior to the interview and verbal consent at the time of the interview. The length of interviews ranged from 24 to 75 min (mean 45 min). Interviews were audio recorded, transcribed verbatim and securely stored in a digital format for subsequent analysis.

Three interviews were identified by the principal investigator to include imposter participants. These participants all used Microsoft Teams as their interview location, did not turn their camera on, and provided inappropriate or inconsistent responses, including incorrect descriptions of services and locations in Queensland, consistent with descriptions of imposter participants reported in the literature [26, 27]. This prompted review of Qualtrics expression of interest forms, which revealed IP addresses located outside of Australia. Data collection was paused, and strategies were implemented to minimise the risk of further imposter participation.

Mitigation tactics included removing recruitment flyers from publicly available community Facebook pages, restricting participation to expressions of interest originating from Australia IP addresses, and incorporating screening processes to verify participant eligibility and contextual accuracy. The interview guide included eligibility confirmation questions, as well as location‐ and experience‐specific questions that would be difficult to answer without genuine lived experience. Upon recommencement of interviews, all Microsoft Teams participants used cameras and interviews conducted via telephone were carefully monitored for any recognised red flags [26, 27].

Following implementation of these strategies, no further suspicious interviews were identified and interviews continued until thematic consistency emerged. The three interviews deemed likely imposter participation were excluded, resulting in a final sample of twenty‐one participants. Gift vouchers were provided to all participants in appreciation of their participation.

### 2.4 Data Analysis

Interviews were inductively analysed, allowing participants' experiences to be constructed from the data. AM and BG independently coded five transcripts in NVivo to cross verify interpretations, reduce bias and increase confidence in the results and thus ensure intercoder reliability [28, 29]. Following this, codes were organised and examined thematically [22, 23]. Coding and theme development then proceeded iteratively, with new transcripts informing and refining emerging themes.

To support a structured interpretation of participant experiences, the Warwick Patient Experiences Framework (WaPEF) consisting of seven evidence‐informed dimensions of patient‐centred care was applied [30]. After themes were established, they were mapped to the WaPEF to contextualise findings and strengthen their applicability for developing patient‐focused recommendations to enhance fertility care. The WaPEF was modified by adding an eighth dimension to capture rural fertility care experiences, and the dimensions were reordered to reflect the fertility journey, in response to the emergence of a previously unrepresented theme of privacy and confidentiality in rural communities. The dimensions of WaPEF are defined in Table 1 to illustrate the relevance to women's experiences of rural fertility care.

**TABLE 1:** Modified WaPEF for dimensions of Women's experiences of accessing fertility care while living in a rural or remote community [30, 31].

Modified WaPEF for rural fertility challenges		
Dimensions	Narrative	Subtheme in results
Information *Based on dimension 6 (Information) of WaPEF*	Information about infertility, when to access care and how to access care is important to enable self‐care and active participation. Certain sources of information in rural areas may be less available than metropolitan areas, including health professional support and peer support. Quality of information from some sources may be inaccurate. Lack of such information can impact the ability to make informed choices	3.1.1 3.1.2 3.2.1
Access to quality fertility care *Based on dimension 4 (Continuity of care and Relationships) of WAPEF*	Accessing quality fertility care including the availability of services, trust in health care professionals, coordination of logistics, barriers to access including distance and financial capacity	3.1.3 3.1.4 3.1.5 3.1.6 3.2.2
Patient as an active participant in accessing quality fertility care *Based on dimension 1 (Patient as active participant) of WaPEF*	Reflects the patient as an active participant in their fertility care, co‐managers of their fertility care and use of services; responsible for participating in choices around what fertility care to access and empowerment	3.1.2 3.1.7 3.2.3
Communication effectiveness *Based on dimension 5 (Communication) of WaPEF*	Effective and appropriate communication methods to ensure that the person's choice to travel is restricted to when necessary. Referrals and results are communicated effectively to necessary personnel between health services to avoid delays to treatment	3.1.8 3.2.4
Health services delivering fertility care appropriate to rural patients *Based on dimension 2 (Responsiveness of services—an individualised approach) of WaPEF*	Availability of services that accommodate the needs of rural patients including limiting travel, accessing services locally (where appropriate) and involving partners (where possible)	3.1.4 3.1.6 3.1.9
Lived experience of accessing fertility care while living in a rural community *Based on dimension 3 (Lived experience) of WaPEF*	Women experiencing fertility challenges can experience a difficult and stressful journey. Experiencing this while living in a rural or remote community creates additional challenges, that are often interrelated and act as potential barriers to accessing fertility care and maintaining a family, work and social life	3.1.10
Confidentiality and privacy in a rural town *Dimension 8 (additional dimension specific to fertility care in rural and remote communities)*	Consideration of the difficulties in maintaining confidentiality and privacy particularly regarding the sensitivity of infertility. This was especially relevant in the workspace due to leave required to access ART when living in rural areas	3.1.11 3.1.12
Support *Based on dimension 7 (Support) of WaPEF*	Support for patients and partners from health professionals through to family, friends and work has characteristics and accessibility challenges for those living in rural areas	3.1.13 3.2.5

Abbreviation: WaPEF, Warwick Patient Experiences Framework.

### 2.5 Reflexivity

Qualitative research is shaped by the researchers involved, each bringing their own background, experiences and worldview to the study [32]. This project was conducted by a team of female academics, health professionals and researchers, who represent diverse personal experiences, choices and perspectives related to fertility. The primary investigator (AM) is a PhD candidate, registered pharmacist with direct insight into the challenges health professionals face when delivering fertility‐related care in these communities. ST, EA and BG further strengthen the team through their roles as health professionals, academics and researchers committed to advancing healthcare service delivery, especially for women in rural and remote Australia. During this study, the researchers remained mindful that shared gender identity could shape data collection and interpretation, and hence reflexive practices were employed throughout analysis to consider and manage potential influences on interpretation. Together, the research team recognises the personal nature of fertility and is motivated to better understand the experiences of women in rural locations.

## 3 Results

Participants' residential addresses at the time of receiving fertility care were classified as MM3–MM7 under the Modified Monash Model, meeting the study's criteria for rural and remote participants [33]. Participants (43%) currently accessing fertility care, with 57% having previously received care, including two pregnant at the time of interview, are labelled as P1–P21 with additional characteristics outlined in Table 2.

**TABLE 2:** Participant characteristics.

Rural/remote category (MM) [participant numbers]	Currently accessing care—time since starting [participant numbers]	Previously accessed care—time since last accessed [participant numbers]
3 [1]	N/A	5–6 years [1]
4 [8]	< 1 year [2]	< 1 year [1]
	10+ years [1]	1–2 years [2]
		3–4 years [1]
		10+ years [1]
5 [4]	3–4 years [1]	3–4 years [1]
	7–8 years [1]	5–6 years [1]
6 [4]	1–2 years [1]	< 1 year [1]
	3–4 years [1]	3–4 years [1]
7 [4]	3–4 years [1]	1–2 years [1]
	5–6 years [1]	5–6 years [1]

Abbreviation: MM, Modified Monash Model.

Two main themes, and 18 sub‐themes emerged from the data. The main themes consisted of: (1) the experiences of participants in accessing and receiving fertility care whilst living in a rural area and (2) participants' perspectives on how to improve fertility care experiences in rural areas.

### 3.1 Theme 1—Experiences of Participants Accessing and Receiving Fertility Care

#### 3.1.1 Access to Information: ‘How Do You Get Cracking When They Don't Give You the Information?’

Participants narratives reflected different levels of awareness of when, who, and how to access advice when experiencing fertility concerns. Underpinning this was a consistent message of infertility being considered a ‘taboo subject, people don't talk about it’ (P1). Limited fertility discussion among rural people was considered to contribute to a lack of knowledge about fertility challenges, particularly infertility prevalence (Table 4 quotes 1a,b). Most participants had difficulties accessing advice, suggesting ‘when we're actually trying to have a baby, the information's just not there’ (P2).

Those describing accessible information spoke of utilising online platforms such as ChatGPT, TikTok, podcasts, Google searches and Facebook infertility forums to access information (Table 4 quotes 1c,d). Information sought ranged from ‘things to explore before seeing a fertility specialist’ (P3) through to ‘advice on the best ART fertility clinics’ (Table 4 quote 1e); however, the reliability and usefulness of information was questioned (Table 4 quote 1f). All participants had consulted their general practitioner (GP) for fertility advice, and some received guidance from friends or family (Table 4 quote 1g).

Consulting a local GP and having an established relationship with them was broadly acknowledged as challenging. Most reported wait times of at least 3 weeks extending to ‘six to eight weeks for an appointment to see a GP here’ (P4). Continually changing GPs decreased participants’ comfort in discussing fertility challenges and seeking support (Table 4 quotes 1h,i).

#### 3.1.2 Choices and Informed Decisions Around ART: ‘Where Can We Go?’

Some participants felt that information provided was insufficient for making informed decisions about treatment options. This included uncertainty around ‘how much of the IVF process can they do remotely and when do you need to go to somewhere else, and where?’ (P7). Others highlighted limited transparency around IVF success rates, noting that, ‘you don't really understand the lack of success that IVF has’ (P6).

Participants perceived having fewer choices in their fertility care, particularly ART, than those in metropolitan areas (Table 4 quote 2a), including clinic selection, access to information, GP support, treatment options, use of donor eggs/sperm/embryos, and the ability to take time off work (Table 4 quotes 2b,c). While some participants recognised that limited choice is part of living rurally, many felt that improvements were possible (Table 4 quote 2d).

Treatment costs and transparency concerns were linked to information being generally inaccessible (Table 4 quote 2e). Several participants were unaware that reduced‐cost clinics existed (Table 4 quote 2f) and one participant described secrecy around fees, stating, ‘the nurse wouldn't even say it aloud. She just pointed and underlined it. $24,000’ (P7).

#### 3.1.3 Financial Impacts: ‘Can We Cut Those Travel Fees Out?’

The costs involved in accessing ART fertility clinics were not limited to treatment. Although government travel subsidies were available, they were reportedly restricted to ART fertility clinics in closest proximity (Table 4 quotes 3a,b) and often only partially covered travel expenses (Table 4 quote 3c) [34]. The participants' narrative reflected that their ability to access fertility care, both initially and subsequently, and the type of treatments they could access was limited by financial capacity (Table 4 quotes 3d–f).

#### 3.1.4 Distance to Services: ‘The Distance Makes It Even Harder’

**FIGURE 1:**
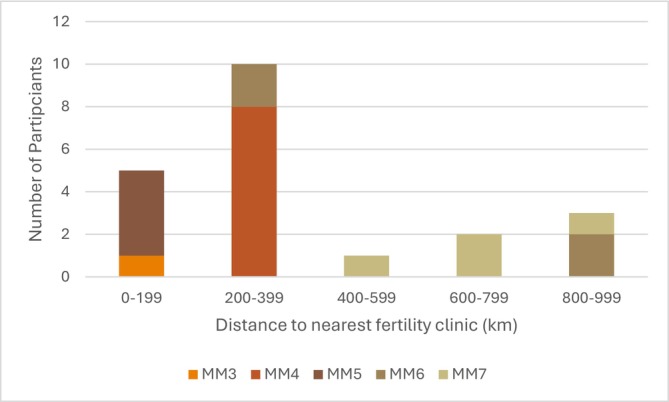
Distance by road between participants' residential address and the closest ART fertility clinic (km).

ART fertility clinics were accessed by just over three quarters of participants, with five preferring not to undergo IVF or to try other treatments first (Table 4 quotes 4a,b). Outside of GPs, some participants accessed fertility care locally from a range of practitioners, including acupuncturists, naturopaths, pharmacists, psychologists, sonographers, phlebotomists and counsellors. To access an ART fertility clinic, however, the shortest distance by road was 47 km (MM5 participant), with most required to travel over 200 km (see Figure 1).

Participants did not always attend the ART fertility clinic closest to their home, with many attending multiple clinics over time. Sixteen participants collectively attended thirty‐eight ART fertility clinics, with the distances travelled shown in Table 3.

**TABLE 3:** Distance by road between participants' residential address and the ART fertility clinics attended.

Distance travelled to ART fertility clinics	Number of clinics MM3 participants	Number of clinics MM4 participants	Number of clinics MM5 participants	Number of clinics MM6 participants	Number of clinics MM7 participants	Total clinics accessed by all participants
0–199 km	2	0	4	0	0	6
200–399 km	0	4	1	2	0	7
400–599 km	0	1	0	0	1	2
600–799 km	0	1	0	0	0	2
800–999 km	0	6	0	3	2	11
≥ 1000 km	0	4	3	0	4	11
All	2	16	8	5	7	38

Abbreviations: km, kilometres; MM, Modified Monash Category.

Choice of clinic was based on proximity to family or friends, who could provide accommodation and support (Table 4 quotes 4c,d), differing expertise of services (Table 4 quote 4e), reduced‐cost options (Table 4 quote 4f), and concerns that the nearest specialist was not suitable for a same‐sex couple (Table 4 quote 4g). As one participant noted, ‘for IVF, you have to choose a doctor that suits and works with you, it's demanding and intimate’ (P6).

**TABLE 4:** Exemplary participant quotes related to theme 1: Experiences of participants in accessing and receiving fertility care whilst living in a rural area.

Subthemes and quotes	
**Access to information: ‘How do you get cracking when they don't give you the information?’**	
1a	‘As a society, no one talks about them [fertility challenges], none of us do, the information's not there’. (P15)
1b	‘And I had no idea that miscarriage was so common. I didn't even know my mum had a miscarriage’. (P16)
1c	‘I think they were on a podcast or something once. She'd had a baby, and she'd listed all the prenatal she had taken. These are the ones. Honestly if it's something I don't have to research, I'm happy to do’. (P7)
1d	‘Since this second transfer, we've done some ChatGPTing and looking at TikTok’. (P1)
1e	‘And so, I was recommended by another lady who lives in [MM4 location] to go to [Fertility Specialist]’. (P6)
1f	‘You turn to Google and there's just that much information, but it's not Queensland based’. (P3)
1 g	‘I did that whole process for 6 months, taking vitamins and not drinking out of plastic and all that cause a friend said she done that’. (P14)
1 h	‘But you know, some women might not necessarily feel comfortable if they're seeing a different doctor every time that they can't even talk to them about that’. (P17)
1i	‘It's so hard cause you can never really get the same GP’. (P11)
**Choice and informed decisions: ‘Where can we go?’**	
2a	‘Whereas Brisbane, you've got everything at your fingertips and if you're not happy with how someone's treating you, you've got options to shop around and more knowledge on everything with the specialists down there’. (P1)
2b	‘[MM4 town], where everyone knows everybody. How do you meet people? How do you meet people who want to donate to you?’ (P6)
2c	‘You have access to so many more options, the stress levels would be lower. Up here, you're very restricted’. (P1)
2d	‘When we moved here, we didn't expect to actually be going through this’. (P3)
2e	‘It felt like they were pushing IVF but not giving any information on when the **** do you start? And where do you go? And even if there is a travel allowance you're entitled to. Because IVF's a big cost in itself, let alone forking out [spending money] for travel and accommodation’. (P5)
2f	‘And there's no like information that bulk billing clinics exist’. (P7)
**Financial impacts: ‘Can we cut those travel fees out?’**	
3a	‘So, we ended up going to the [MM1 location]. But because [MM2 location] is our closest clinic, no patient travel or anything, none at all’. (P14)
3b	‘And that was the big thing, you can't choose. You must go to your nearest one, otherwise patient travel doesn't pay’. (P14)
3c	‘You get subsidies, but it doesn't cover cost. I think you get $60.00 a night. But to book a hotel in [MM2 location] it is $250. So, it's some help, but you're a lot out of pocket still’. (P13)
3d	‘And I can afford it, but there's other people that can't. And that, is a barrier for accessing fertility care’. (P1)
3e	‘If we couldn't have afforded it, it wouldn't have happened. It's just not available for everyone to be able to go to a fertility clinic’. (P11)
3f	‘We'll just do whatever it takes, whatever it takes. We'll do it. Because we didn't have finance limiting us’. (P6)
**Distance to services: ‘The distance makes it even harder’**	
4a	‘We still are not ready for IVF. According to the specialist, it's still the best way to have the best outcome’. (P12)
4b	‘I just want to try it out naturally, for now. I feel like there would be more options rather than just go straight to IVF’. (P2)
4c	‘We were lucky that we had family. We could stay at their house’. (P17)
4d	‘I went there because my family lives at [MM1 location] and so I had support. I didn't know anyone in [MM2 location] and so I went [to MM1 location] because, it's all new, a bit scary’. (P6)
4e	‘The expertise that was needed wasn't available at that clinic’. (P6)
4f	‘Even if you want to access the bulk bill of side of things like you've still got to travel to [MM1 location]’. (P1)
4 g	‘My GP at the time said that he's a fantastic doctor and obstetrician, but she just wasn't sure where he sat on same sex couples because I believe he's [religious affiliation]. So, she said I'm not sure, but I would just hate for you to have a negative experience’. (P10)
**Away from home: ‘Just logistically a nightmare’**	
5a	‘I just went home after each appointment because we didn't have too far to travel’. (P18)
5b	‘Often go to [MM1 location] for 2 or 3 weeks because I didn't ovulate till day 24. So already, that's 6 more days. So, then it just becomes longer and longer’. (P6)
5c	‘That's a logistical nightmare. And work as well. Someone comes up and look out for the chickens. You've got to look at all these little things too. You can't just leave it all and say see you later’. (P5)
5d	‘It's just the effort of putting in leave and travelling and taking my son with and my partner and accommodation and everything. It's a massive procedure travelling wise’. (P12)
**Fertility care at home: ‘Access to things is a lot harder’**	
6a	‘The time it took trying to book an appointment. And the cost. That was really challenging’. (P3)
6b	‘Another expense is if I was having my scans in [MM1 location] at the clinic, they would be just included in the cost of the treatment. But because we're having them locally, they're all out of pocket’. (P10)
6c	‘And just because they don't trust the rural sonographer. I've got to pay $500 in flights for 1 day to come in and do a scan and come back’. (P14)
6d	‘And then pathology was closed for the weekend, so I had to wait till Monday. I had to continue meds that I didn't need to be on, and it was just a big emotional thing’. (P1)
6e	‘General access to things is a lot harder, it's an extra 2 days to get results’. (P1)
6f	‘You can only have two blood tests, but we need you to have 10 blood tests and only the 1st 2 we're paying for. So, you have to pay for the rest. Because I can't physically get to that clinic’. (P6)
6 g	‘We had to have another semen analysis through [ART fertility clinic], they're not trusting [pathology name] and [pathology name] and [pathology name] and they want to do it in house’. (P3)
6 h	‘We had to get to [MM2 location] because we don't have a basic collection that are able to look at it immediately. It means going to [MM2 location], which was quite a big deal for him. That just added a really stressful, hard element and he literally had to get on a plane, fly down’. (P13)
6i	‘And then people are saying, can you just organise your Courier? I need that information, and it's going to cost another $500 that I didn't tell you about’. (P5)
6j	‘If we had done it at the time as was planned, and as I'd agreed to, we may have been further ahead by now. In the position of seeing the specialist in Brisbane 2 years ago. So, there's a bit of frustration there’. (P3)
**Self‐management: ‘You feel like you've got some control’**	
7a	‘I have seen a psychiatrist through the IVF. Which I pay for myself. It wasn't recommended by the clinic or anything. I sourced her’. (P6)
**Communication: ‘Do they know where we live?’**	
8a	‘I know you have to take time off. Look, there is no expectation (of you to come down) we can do this all over the phone and over the zoom. He was really good, whereas you would get other doctors that would just not even consider that’. (P10)
8b	‘The office ladies still don't get it; the admin staff still don't get it. They don't get that my husband and I are not in the same places at the same time’. (P6)
8c	‘That might just be the service delivery of the fertility clinic, so what they do is in that specific location versus how we can make our service adaptable for people who can't travel here or can't be here for the whole period of time’. (P9)
8d	‘Aside from the procedures, everything was done by telehealth or video conference’. (P3)
8e	‘IVF is going to be stressful enough as it is. I don't need to be chasing everybody because there's no communication’. (P5)
8f	‘I hadn't heard from anyone, so I ring up [MM6 hospital]. Received nothing. I ring up [M7 GP clinic], they should have been sent through the referral. Well, [MM6 hospital] say they haven't got it. OK, we'll send it through. Not sure what's happened. Ring [MM6 hospital]. Nothing. Ring [MM7 GP] get told a completely different story’. (P5)
8g	‘I don't know what happened with the ultrasound. That's gone missing for a couple of days now, and because it all is also timed, sensitive and if my hormones aren't at the levels that they should be at, then we can't make the changes with medication if I'm remote’. (P9)
**Partner inclusion: ‘He had to work. Someone had to pay for all of this’**	
9a	‘Since we've transitioned over to the [MM1] specialist, he feels more involved in it now’. (P3)
9b	‘Because we've both been having procedures, you'd assume we both would have been in communication. So, it's really interesting that she still wasn't included’. (P10)
**Impact of rural infertility life: ‘I don't think they understand’**	
10a	‘Financial stress. Time away from your community. It was a lot’. (P16)
10b	‘I was very disappointed in the specialist with the lack of follow up, the lack of even diagnosing. That it wasn't hyperstimulation. But then I lost an ovary out of this because he didn't bother’. (P19)
10c	‘Maybe if I'd be in [MM1 location], I would have got the proper medical treatment and not had to be flown to the hospital in [MM2 location]. I mean, it's all maybes, but the expertise around IVF wasn't there’. (P6)
10d	‘Because you're choosing to have IVF, or you need to have IVF, and you should have to pay for it’. (P6)
10e	‘First is privacy and confidentiality for me, and minimising shame. And maybe some emotions that might make me feel uncomfortable in relation to that. But then the second part is because I know them’. (P9)
10f	‘It's a dictatorship type naughty school kid thing. You waited too long, or you did this, or you did that too old, or you're pushing’. (P5)
10g	‘I didn't talk to many people about it in the community either because everyone was having babies, and it was difficult to think what's wrong with me. Why can't I keep it’. (P16)
**Privacy: ‘How do I keep this to myself at work?’**	
11a	‘If you have sick leave and you don't want to tell your manager, it's hard. I got a medical certificate for it, but it's still said, Doctor [MM2]'s office, obstetrician’. (P17)
11b	‘Medical certificates, I have asked my practitioner, can we write a letter? But can we do it without a signature lock? It's a small town’. (P9)
11c	‘If you didn't have to tell them why you're going away so often, you would have a bit more privacy’. (P8)
11d	‘I tried to keep it separate from work but now my manager knows because I have to put in leave, and he needs to approve it’. (P12)
**Support: ‘Who do you talk to?’**	
13a	‘No local support, counselling, or anything. There's no point of call if you're have having difficulties with fertility’. (P11)
13b	‘That's where counselling would be beneficial to people because, I feel like such a failure. And you don't want to, tell anyone that. There is no one else to talk to apart from your partner’. (P11)
13c	‘The [MM1] specialist has been really great and has flagged that if you need any referrals, please let me know and he's happy to do it’. (P3)
13d	‘It was private. People don't understand, or you don't tell everyone what you're going through, especially with your friends who've had it easy’. (P15)
13e	‘My workplace is quite supportive in that they let me work remotely from home’. (P9)
13f	‘We're so lucky we've got fantastic employers because we're going to get the medication here’. (P10)
13g	‘I have a great team that's very supportive. So, I have the flexibility to do that. It's just challenging’. (P13)
13 h	‘I wouldn't say that he's ever been offered support. He hasn't expressed a need for it, but I know that it's gotten harder on him. So, I think it wouldn't hurt if he spoke to someone about it’. (P3)
13i	‘He hasn't expressed a desire but he's not very likely to chat to his mates about it. Like you chat to me and there's only so much I can help, cause I'm going through it too’. (P3)

Abbreviation: MM, Modified Monash Category.

#### 3.1.5 Being Away From Home for ART: ‘Just a Logistical Nightmare’

Three participants lived near enough to drive home after most appointments; however, others were required to spend time away from home or undertake two flights in 1 day (Table 4 quotes 5a,b), impacting partners, families, and workplaces. Participants reported ‘it makes me more nervous to be away’ (P9) and ‘when I stay in accommodation, I feel unfamiliar or uncomfortable’ (P6). The dates participants were required at a treatment location could be uncertain, which meant organising flights and accommodation, time off work and care for children or animals with ‘only have a couple days’ notice’ (P10) (Table 4 quotes 5c,d).

#### 3.1.6 Fertility Care at Home: ‘Access to Things Is a Lot Harder’

For participants receiving care through ART fertility clinics, some services e.g., ultrasounds could be performed locally, however, access was limited. Reported challenges included long wait times for appointments (Table 4 quote 6a), additional costs for scans performed outside of the clinic, and some specialists' lack of trust in externally performed services, leading to repeat scans and further travel (Table 4 quotes 6b,c).

Similar challenges occurred with pathology testing conducted outside of clinics. Participants reported difficulty accessing weekend testing (Table 4 quote 6d), delays in receiving results (Table 4 quote 6e), extra costs (Table 4 quote 6f), and issues with inaccurate samples (Table 4 quote 6g). This led to some participants spending extended periods away from home. (Table 4 quote 6h).

Participants also raised broader concerns about the quality of rural fertility care. These included increased expectations to organise their own services, changes to procedures that restricted available treatment options at certain clinics, and potential delays in care due to perceived lower quality of service (Table 4 quotes 6i,j). One participant summarised, ‘you're kind of stuck with what you've got here’ (P3).

#### 3.1.7 Self‐Management: ‘You Feel Like You've Got Some Control’

Rural participants described having to manage much of their own fertility care. Taking an active role, ‘doing the best thing that you can, to give yourself the best chance’ (P11) was considered important to maintain control. Some participants independently sought fertility care services outside of what they considered the standard medical approach, such as mental health care, dietitians, acupuncturists, and naturopaths, while others were unaware these options existed (Table 4 quote 7a).

Living rurally added practical and emotional strain, with ‘little things adding stress in an already a difficult situation’ (P1). Despite having limited influence over treatment and outcomes, some participants reported choosing to engage in fertility care outside of ART such as healthy eating, exercise, or ‘booking this acupuncture appointment and hoping for the best’ (P3), helped them feel empowered. These actions provided a sense of agency: ‘It's not just medication or another round of blood tests…. knowing about those things is just empowering’ (P3).

#### 3.1.8 Communication: ‘Do They Know Where We Live?’

Some ART fertility clinics had processes and staff that reflected an understanding of distance‐related barriers and provided clear expectations regarding travel ‘He said: I'll be really mad if I see you guys down here for anything other than the procedures, I know how expensive it is’ (P10) (Table 4 quote 8a). For participants required to travel for appointments, most health services offered telehealth as an option, however, this was not consistent for all participants (Table 4 quotes 8b–d). Although ‘we prefer face‐to‐face’ (P12), most considered it is ‘better when you can do it in your own home, so you don't have to be driving to a different town’ (P2). Other methods of communication included ‘they email us all the blood tests and stuff we have to do’ (P7) and acknowledgement that telehealth increases access as ‘there's no way I could have done IVF if I didn't have telehealth’ (P6).

Inadequate communication between health services and patients was reported by some participants leading to increased stress and feelings of responsibility (Table 4 quote 8e). Coordination challenges extended beyond those undergoing ART. Several participants experienced communication breakdowns between rural health professionals and services, including ‘referrals not being received (Table 4 quote 8f), missing results (Table 4 quote 8g), and incorrect information being provided for accessing care’.

#### 3.1.9 Partner Inclusion: ‘He Had to Work. Someone Had to Pay For All of This’

Most participants described difficulty in the ability of partners to attend appointments and procedures due to location. When able to attend, some ART fertility clinics made efforts to include partners in procedures, (Table 4 quote 9a) however in other circumstances, participants felt ‘the treatments were all geared towards females, until the medical diagnosis says it's yours [male factor infertility]’ (P7). Discussion of male partner inclusion mentioned a perception of being on the periphery ‘he just sits on the edge and sees the outcome, without being part of the process’ (P13). However, female partners also had similar feelings of ‘she felt so detached from everything’ (P14) and not being involved even when they were both undergoing procedures (Table 4 quote 9b).

#### 3.1.10 Impact of Rural Infertility on Life: ‘I Don't Think They Understand’

Living with fertility challenges in a rural location impacted many areas of participants' lives, ‘our financials, our friendships – there's been nothing in my life that has not been impacted by IVF’ (P6). Rural impacts went beyond financial and time issues (Table 4 quote 10a), with rural culture contributing ‘it is so hard, expensive and isolating, especially for people in rural remote areas, they just don't talk about it’ (P11). For one participant, her fertility journey made her question living in a rural location, ‘the whole thing made me go I don't want to be living out here when I do have children. I don't want to live in this rural community’ (P12).

For some participants, the inability to stay extended periods of time close to ART fertility clinics potentially impacted their health. One participant was warned ‘you could get quite sick. He would have preferred us to stay, but we weren't in a position and had to get back to [MM4 location]’ (P14). Health impacts were described as post‐treatment ovary damage, and ovary loss due to post‐treatment complications occurring in a rural location (Table 4 quotes 10b,c).

Feelings of judgement and shame were shared including perceptions of fertility being considered a socially constructed need (by others) (Table 4 quote 10d), difficulties with confidentiality, internal feelings (Table 4 quote 10e), and attitudes of health professionals (Table 4 quote 10f). Experiencing fertility challenges was socially isolating for many participants, who spoke of difficulty making or maintaining friendships and socialising due to the emotional impact of fertility challenges (Table 4 quote 10g).

#### 3.1.11 Privacy: ‘How Do I Keep This to Myself At Work?’

Strong reflections of difficulties maintaining privacy in the workplace were consistently delivered, stemming from medical certificates with doctor's names and locations on them (Table 4 quotes 11a,b) through to the storing or receiving of medications at work, and for most participants, the requirement to ask for extensive leave, sometimes with little notice (Table 4 quotes 11c,d). Some participants choose to tell certain colleagues, others did not. Irrespective of whether their fertility journey was shared with colleagues, ‘you knew that everyone was thinking it. I just wanted to be able to deal with it ourselves, but when you're away for extended time, it just becomes obvious’ (P14).

#### 3.1.12 Conflict of Interest and Confidentiality: ‘We All Go to the Same Parties’

In a small town, blurring of personal and professional lives was described as contributing to confidentiality concerns ‘So, it's like I know he knows, and he knows I know he knows, but who else knows?’ (P9). Some participants were unable to consult a local health professional due to ‘it being a conflict of interest [because I work in this area]. I'm not eligible to seek support from my own organisation’ (P13). Others felt ‘uncomfortable because I don't necessarily want to see someone I'm also going to see at work’ (P2).

#### 3.1.13 Support: ‘Who Do You Talk To?’

For most participants, formal mental health and emotional support had not been offered either locally or through ART fertility clinics (Table 4 quotes 13a,b). Some clinics proactively referred patients for counselling (Table 4 quote 13c); however, ‘it was like 2 weeks till she could talk to me. That period was really, really dark’ (P14). Although some participants were supported by friends and family, not all sought care from these networks (Table 4 quote 13d).

Maintaining privacy was considered especially important to some participants and their selectivity of support sources was highlighted, ‘I don't speak usually…I have great friends so the ones that are meant to know, know’ (P13). Although participants shared issues of maintaining privacy, most described supportive workplaces. This included support in working remotely (Table 4 quote 13e), receiving medication deliveries (Table 4 quote 13f) and allowing flexibility for time away from work (Table 4 quote 13g).

Several participants thought that support for their partner could be beneficial, although in general males did not express to their partners a need for counselling (Table 4 quotes 13h,i). The participant's perception of partner experience included ‘as a man he felt quite vulnerable. Helpless. Not in control. All the things that men hate the most’ (P11) and ‘she even said it's all very clinical, I feel like I'm not part of this whatsoever’ (P14).

### 3.2 Theme 2—Participants' Perspectives on How to Improve the Fertility Care Experience in Rural Areas

Participants' recommendations to improve the fertility care experience in rural areas were themed to the WaPEF dimensions, and further sub‐themed to provide detail and clarity. Participants' recommendations were their own thoughts, generated at the time of the interview, and not selected from a list or directly prompted. Table 5 summarises the frequency of participants discussing themed wants and needs, indicating the breadth of perspectives represented. These counts reflect the number of unique participants who mentioned each ‘want’ at least once and are provided to illustrate the prominence of their concerns.

**TABLE 5:** Frequency of participants identified wants and recommendations for improving rural fertility care.

Modified WaPEF for rural fertility challenges dimensions/subthemes	Number of participants *n* (%)	Example quote
**3.2.1. Information**		
Treatment options/pathways for fertility care (more information available)	9 (43)	‘Pathways. What your options are. This is what the processes are. This is who you can talk to about it. Give you a bit more choice and understanding’. (P5)
Understanding of the requirements of undergoing ART to make informed decisions	6 (29)	‘I'd like to see more acknowledgement and information on the impact that it has on people physically and mentally’. (P6)
Awareness and normalisation of fertility challenges	5 (24)	‘And it might be just to talk about it more openly. Normalise it’. (P11)
Travel (logistics)—booking accommodation, public transport	4 (19)	‘If there was someone especially for rural (people), just to get your head around the logistics. How often am I going to have to travel? What does that look like? To understand about the travel, to understand how urgently you may have to do it it's the start of a conversation, and then you might be able to get some direction from that’. (P10)
General fertility knowledge	2 (10)	‘If there was actually someone you could ask for general guidance around it [infertility]’. (P10)
Men's fertility education	1 (5)	‘Even if that means you go around and run education programmes in men's sheds, whatever it is to access men’. (P6)
**3.2.2. Access to quality fertility care**		
Services available locally (pharmacy/medication availability, counselling, pathology, imaging, hospital)	9 (43)	‘The clinic should be saying to the people in a rural place, you can go to your local clinic, you can go to your local hospital or your local sexual health clinic. And the nurses there will help you [inject medication]. Because I was never told that there was an option like that’. (P17)
Local clinic/contact/hub (information and advice) women's health/fertility	8 (38)	‘Just someone who is there, so you don't have to wait months. That you can get referred to and you can have a point of contact. Like a hub or something that could focus more on Women's Health’. (P4)
Increased accessibility to imaging (ultrasounds)	8 (38)	‘Accessibility to practices like the ultrasound technicians, especially when there's so many of them [ultrasounds]’. (P3)
Outreach clinics (nurses and/or fertility specialists)—initial visits and follow up	6 (29)	‘If they did outreach work, those check‐ups and the initial scans or checks, blood tests, that kind of stuff would be great’. (P13)
Increased accessibility to pathology	5 (24)	‘To potentially have somewhere where you can even drop into and go help me coordinate this blood test’. (P14)
To be fully covered (financial accessibility)	4 (19)	‘That could all [travel costs] be fully covered’. (P13)
Reduced fee/subsidised assisted reproductive technologies for those living in rural/remote areas	4 (19)	‘Why aren't rural and remote people who are going through IVF subsidised either through Medicare [government support] or however many scans you need is subsidised. That's part of the reason I've had to uproot myself’. (P6)
Multidisciplinary team	3 (14)	‘Having an interdisciplinary or multidisciplinary team working together would potentially be more useful and come to a consensus. Instead of one individual here, one individual there that have got their expert knowledge in some areas but not the whole picture’. (P9)
Scans—not having to pay for extra away from clinic	3 (14)	‘You only get 2 scans because the rest of them are expected to be done in clinic. That's not realistic because I can't physically get to that clinic’. (P6)
Blood tests—not having to pay for extra away from clinic	3 (14)	‘That would be great if that was fully covered, because that would take a lot of the stress out of it knowing that your bloods are covered financially’. (P13)
Classes	3 (14)	I wish they had classes or an info session even’. (P2)
GPs trained in fertility care	2 (10)	‘I just wish more GP's realised the importance of it and had more knowledge. It's very lacking and I wish they had a protocol of how to refer people’. (P2)
Accessing fertility care rights for LGBTQ+	1 (5)	‘I'd like to see greater rights [for same sex couples] generally around IVF and understanding’. (P6)
Access to surrogacy bank	1 (5)	‘For gay and lesbian couples to have greater access to a surrogacy bank. You know so that you're not sourcing sperm from a Facebook account’. (P6)
**3.2.3. Patient as an active participant in accessing quality fertility care**		
To cover travel other than closest clinic (choice in clinic to access)	4 (19)	‘The big thing is you can't choose. You must go to your nearest one, otherwise patient travel won't pay’. (P14)
Awareness of availability and how to access services	2 (10)	‘Information on things like patient travel, to find out about that from somewhere’. (P19)
**3.2.4. Communication effectiveness**		
Telehealth (video) for all appointments not requiring face to face (all health professionals)	6 (29)	‘There's not even telehealth. So that would be a big improvement in my opinion’. (P6)
More empathetic communication	3 (14)	‘I was pretty upset, and I was crying. And she was like, what are you crying about?’ (P20)
Respectful/non‐judgemental communication	2 (10)	‘Not being made to feel that you're too old. And not being treated like a school kid. And that they follow through with what they say they're going to do’. (P5)
**3.2.5. Support**		
Mental health support (local and telehealth) patient	11 (52)	‘A counsellor who's a professional in that area, 100% could talk you through those feelings and normalise it as a result of having experience in that space’. (P11)
Indigenous culture	4 (19)	‘Because as an Indigenous person, when we have women's business, it's circles, women sharing that common theme and thread. So, it would've been nice for me talking one on one to a counsellor, but I'd be thinking, does she have the same lived experience as me to know what I'm talking about?’ (P9)
Mental health support (partner)	2 (10)	‘And support to husbands, because if it's not a male factor infertility thing they get pushed to the side. They're going through the same thing, and they feel more helpless because they can't control it. They can't take the burden of it all’. (P1)
Neurodivergent	1 (5)	‘For my retrievals, I had to go under because of sensory issues. So, who's going to pick me up from the hospital? There was no one and I needed that’. (P6)

Abbreviations: GPs, General practitioners; IVF, in vitro fertilisation; LGBTQ+, Lesbian, gay, bisexual, transgender, and queer (or questioning), plus additional identities; WaPEF, Warwick Patient Experiences Framework.

## 4 Discussion

Women who received fertility care while living in rural Queensland shared their experiences of, and perspectives on ways to improve, fertility care. Living in a rural location was considered to add challenges and complexity to an already difficult fertility‐related life event. Although some challenges were not rural specific, participants considered them to exceed what would generally be experienced in metropolitan areas. Living rurally was considered to impact fertility knowledge and available information, accessibility to services, privacy, and support. Although some aspects are difficult to modify e.g., distance from ART fertility clinics, strategies were proposed to reduce the burden of postcode‐based disadvantage in accessing fertility care.

Rural impacts on fertility care experiences were found to be multifactorial and interrelated, which aligns with other studies of ART accessibility [1, 14]. A previous study of gaps in Australian's fertility knowledge revealed inadequate knowledge of preventing infertility and accessing fertility treatment, potentially restricting reproductive goals [6]. This aligns with our findings of limited infertility knowledge, which was exacerbated in rural areas due to its sensitive and private nature. Limited discussion of infertility among friends and family contributed to poor awareness of how and when to seek help, how common fertility issues were, and increased feelings of isolation and lack of support. Societal taboos regarding sexual and reproductive health are known to vary across cultures and affect access to information and healthcare services. These findings align with participants' narratives and highlight the importance of culturally safe care, warranting further research, particularly among Aboriginal and Torres Strait Islander peoples [8, 35].

Insufficient fertility knowledge contributed to a lack of understanding of fertility care options and their access. Additionally, many participants were not aware that fertility care can include FABM, improving preconception health, and modifying lifestyle factors, which may enhance conception rates and overall wellbeing [7]. Accessing fertility care was further impacted by wait times to see a GP and difficulty establishing rapport or discussing sensitive fertility concerns, attributed to high GP turnover [4, 6].

Although not all participants wished to undergo ART, those that did, reported difficulties with accessibility. For many, their closest ART fertility clinic was in a large regional area, however not all ART fertility clinics provided the same services, limiting choice. This aligns with an Australian study reporting that some ART fertility clinics restrict the services offered in regional locations and lack consistent staffing [1]. Variability between clinics was also evident in the availability of telehealth and flexibility of services related to travel requirements, supported by a study which described inconsistency in telehealth utilisation by clinics [1].

The ability to access ART was described as limited to those who had financial capacity as most ART fertility clinics are private [10]. Although publicly funded and reduced‐cost ART fertility clinics exist, they are in metropolitan areas in Queensland, may have eligibility criteria, and are not necessarily free [15, 16, 17]. In addition, some locally available pathology and medical imaging services incurred extra costs or were not accepted by clinics, meaning participants had to travel to clinic sites instead. This experience is consistent with findings reported in other studies [1].

Apprehension around privacy and confidentiality was shared, particularly from those who worked in health settings. Even for rural women working outside of health, a study described rural health care services as having privacy and confidentiality concerns [3]. Conflict of interest prevented some from accessing certain fertility care services and most discussed significant challenges in maintaining privacy when undergoing ART. This was due to the need to inform managers of treatment as ART requires extended and unpredictable time away from home, considered unique to those in rural areas due to the distance required to travel [14].

Although privacy was difficult to maintain in the workplace, the support given by workplaces in accessing fertility care was generally reported as positive. The provision of professional mental health support has been reported in literature as inconsistent and in need of improvement which was also reflected in this study, with only a small number of participants having been referred to a fertility counsellor [36, 37]. Friend and family support in rural areas was reported as variable, explained by a want for privacy and/or feeling uncomfortable around those with children [8].

### 4.1 Strengths and Limitations

A key strength of this study is its exploration of women's fertility journeys across the broader continuum of care, not focusing solely on ART, providing understanding of how rural locations influence access to and delivery of fertility care. Another strength is the ability to identify gaps across all stages of care, from initial awareness and knowledge through to treatment pathways and support. Additionally, the study is inclusive of all women undergoing fertility challenges, including same‐sex couples.

This study however has limitations. Although the MMM was used to indicate participants' rurality, it does not necessarily reflect actual distance to ART fertility clinics; therefore, it could not be used to meaningfully compare differences in access to fertility services. Another limitation was the suspected presence of imposter participants, which required restricting recruitment advertising; however, the integrity of the study was maintained through early identification of the issue and prompt modification of recruitment procedures. Only female participants were included and while this aligns with the study focus, cultural norms around masculinity and fertility can also shape men's reproductive health experiences and warrant future investigation. An intentional ethical decision was made to limit the collection and reporting of participant demographic characteristics, including age. Although this is a limitation which may reduce the contextual interpretation of the findings, it was considered an important step to minimise participant burden and distress, and to reduce the risk of deductive identification in small rural communities when discussing the sensitive topic of infertility [38]. Finally, as the study was conducted in Queensland, Australia, the generalisability of findings to other rural locations nationally or internationally remains uncertain.

### 4.2 Implications and Future Directions

Participant recommendations on areas that could improve experiences for rural women undergoing fertility care have implications for practice, including greater access to information about fertility care and treatment for those living in rural and remote areas. Greater choice in treatment options and locations, supported through improved patient travel subsidies, government support, and increased local service availability requested, has implications for future service delivery. This could be achieved through telehealth, clearer communication tailored to rural contexts, and better awareness of local fertility‐related services. Specialist counselling that is both financially and geographically accessible, along with culturally safe and appropriate support services, was strongly recommended. A local contact point or hub was recommended as pivotal to accessing information, coordination of services, communication pathways, and emotional support throughout the fertility journey.

Incorporating fertility education into secondary school curriculum, as highlighted in a previous Australian study, as a future direction may enhance understanding of proactive infertility prevention and modifiable risk factors, including delayed childbearing [9]. Training accessible health professionals such as general practitioners, pharmacists, allied health professionals and health workers, could enhance access to reliable, culturally appropriate information such as care pathways, treatment options (e.g., lifestyle modification and FABM), timely access, health promotion, screening of fertility concerns and referral processes. Offering community‐based services in underserved regions may bridge service gaps for rural communities and those with limited transport options which is supported in other findings [8]. To provide further insight into the influence of geographical location, comparative studies on persons' experiences between rural, remote, regional and metropolitan geographical settings is recommended. In addition, addressing recommendations for policy reform related to government financial support, alongside further research into education strategies and training of accessible health care professionals, will be important to guide future directions and reducing inequities in access.

## 5 Conclusion

Although travelling long distances is commonplace for people living in rural areas, disparities exist in the ability to access fertility care based on postcode. For those living in rural or remote areas, accessing fertility care, especially ART, involves many factors coming into play including the knowledge of how to access and financial, time, workplace, physical and emotional capacity. Those living in rural areas report a reduced ability to maintain privacy about seeking treatment and may be limited from accessing care locally due to workplace conflict of interest or not wanting to receive care from someone they know. Although difficult to solve all barriers to accessing fertility care rurally, changes can be made to both policy surrounding and physical provision of fertility care to improve the experience and potential outcomes for many.

## Author Contributions

**Beverley Glass:** conceptualization, methodology, writing – review and editing, formal analysis, supervision. **Emma Anderson:** conceptualization, methodology, writing – review and editing, supervision. **Selina Taylor:** conceptualization, methodology, writing – review and editing, supervision. **Amanda Mackay:** conceptualization, investigation, writing – original draft, methodology, writing – review and editing, formal analysis.

## Disclosure

The authors have nothing to report.

## Ethics Statement

This study was approved by James Cook University Human Research Ethics Committee (H9580) on the 8th of October 2024.

## Data Availability

The data that support the findings of this study are available on request from the corresponding author. The data are not publicly available due to privacy or ethical restrictions.
